# Extended spectrum beta-lactamase and fluoroquinolone resistance genes among *Escherichia coli and Salmonella* isolates from children with diarrhea, Burkina Faso

**DOI:** 10.1186/s12887-020-02342-z

**Published:** 2020-10-03

**Authors:** René Dembélé, Ali Konaté, Oumar Traoré, Wendpoulomdé A. D. Kaboré, Issiaka Soulama, Assèta Kagambèga, Alfred S. Traoré, Nathalie K. Guessennd, Awa Aidara-Kane, Amy Gassama-Sow, Nicolas Barro

**Affiliations:** 1 Laboratory of Molecular Biology, Epidemiology and Surveillance of Bacteria and Viruses Transmitted by Food, Centre for Research in Biological, Food and Nutritional Sciences, Graduate School of Science and Technology, University Joseph KI-ZERBO, 03, BP 7021 Ouagadougou 03, Burkina Faso; 2 Training and Research Unit in Applied Sciences and Technologies, University of Dedougou, BP 176, Dedougou, Burkina Faso; 3 National Centre for Research and Training on Malaria, 01, BP 2208 Ouagadougou 01, Burkina Faso; 4 Institute of Sciences, 01, BP 1757 Ouagadougou 01, Burkina Faso; 5 Laboratory of Bacteriology-Virology, Unit of Antibiotics, Natural Substances and Surveillance of Resistance of Microorganisms to Antimicrobials, Pasteur Institute of Abidjan, 01, BP 490 Abidjan 01, Ivory Coast; 6 Laboratory of Bacteriology-Virology, Unit of Training and Research of Medical Sciences, University Felix Houphouet BOIGNY, 01, BP V34 Abidjan 01, Ivory Coast; 7 Unit of Experimental Bacteriology, Pasteur Institute of Dakar, 36 Avenue Pasteur, BP 220 Dakar, Senegal

**Keywords:** Antibiotics, Resistance genes, *qnr*B, *bla*_OXA_, *bla*_CTX−M_

## Abstract

**Background:**

The emergence and spread of multidrug-resistant gram-negative bacteria (MDR) has become a major public health concern worldwide. This resistance is caused by enzymes-mediated genes (i.e., extended spectrum beta-lactamases) that are common in certain *Enterobacterioceae* species. However, the distribution of these genes is poorly documented in Burkina Faso. This study aims to determine the prevalence and distribution of the resistant genes coding for broad spectrum beta-lactamases and quinolones in rural Burkina Faso.

**Methods:**

Multiplex PCR assays were carried out to detect ESBL-encoding genes, including *bla*_OXA_, *bla*_TEM_, *bla*_CTX-M_, *bla*_SHV_. The assays also assessed the presence of quinolone resistance gene namely *qnrA, qnrB and qnrS* in the quinolone-resistance DEC and *Salmonella* strains.

**Results:**

The Extended-Spectrum Beta-Lactamases (ESBL) resistance phenotype was reported in all the *E*. *coli* isolates (5/5). Cross-resistance phenotype to quinolones (CRQ) was shown by one *Salmonella* strain (1/9) and three *E*. *coli* (3/5). Cross-resistance phenotypes to fluoroquinolones (CRFQ) were harboured by one *Salmonella* (1/9) and carbapenemase phenotypes were detected in two *E*. *coli* strains (2/5). Whilst the *bla*_OXA_ genes were detected in 100% (5/5) of *E*. *coli* isolates and in 33.33% (3/9) *Salmonella* isolates. One strain of *E*. *coli* (1/5) harbored the *bla*_CTX−M_ gene and the *qnr*B gene simultaneously.

**Conclusions:**

This study identified β-lactam (*bla*) and quinolone resistance (*qnr*) genes in multidrug-resistant *E*. *coli* and *Salmonella* spp. in rural Burkina Faso. Our finding which highlighted the *enterobacteriaceae* strains resistance to β-lactams and quinolones are of high interest for adequate management of antimicrobial resistant genes outbreak in Burkina Faso.

## Background

Diarrheal disease is the second leading cause of death among children aged below 5 years [1]. These diseases are especially common in developing countries with poor hygiene and sanitation and with limited access to safe drinking water [2, 3]. In our previous study, the overall prevalence of gastrointestinal infections was 19.7% in children group [4]. These infections are due to bacteria such as *Escherichia coli* and *Salmonella* which remain major contributors to acute enteric infection in children. However, the emergence and spread of multidrug-resistant gram-negative bacteria (MDR) has become a major public health concern worldwide [5]. Extended-spectrum beta-lactamases (ESBL) producing *Enterobacteriaceae* isolates, particularly in *Escherichia coli*, have been frequently reported in recent years at global scale [6–8]. Indeed, ESBL- producing *Enterobacteriaceae* (ESBL-PE) are associated with high morbidity and mortality rates, prolonged hospital stays and increased costs of healthcare[9, 10]. Some studies have shown that ESBL are responsible for producing antibiotic-resistant bacteria strains [11, 12]. The spread of the strains is likely to limit the effectiveness of antimicrobials used to treat the patients suffering from pathogen bacteria such as *Escherichia coli* and *Salmonella* [13–16]. These ESBL-producing bacteria often show resistance to several antimicrobials such as third and fourth generation cephalosporins as well as quinolones and aminoglycosides [17–19]. Although inhibited by clavulanic acid, ESBL enzymes have the ability to hydrolyze third generation cephalosporins and aztreonam [9].

The first ESBL strain which was a *Klebsiella ozaenae* resistant to oxyimino-cephalosporins was discovered in Germany [20]. In addition to β-lactams, fluoroquinolone resistance due to Qnr genes is emerging and this may pose a challenge in treatment of typhoid in future. These genes belong to the family of repeat pentapeptides that are capable of binding to DNA gyrase and topoisomerase IV, and thus protecting them from inhibitory activities of quinolones [21]. The resistance to quinolones (*qnr*A) mediated by plasmids in an isolate of *Klebsiella pneumoniae* was first reported in 1998 from the United States [22]. The excessive use of antibiotics, in particular β-lactams, leads to the selection of ESBL producing strains [23]. However, in developing countries, *E*. *coli* identification and microbial drug resistance tests have been limited by phenotypic methods.

Although several antibiotic resistance gene studies have been carried out in Burkina Faso, these studies have been solely conducted in Ouagadougou and Bobo-Dioulasso’s hospitals [24–26]. Therefore, the objective of the present study was to determine the prevalence and distribution of resistance gene coding for broad spectrum beta-lactamases and quinolones in two remote rural health centres (Boromo and Gourcy). The main economic activities in these communities are subsistence farming, animal husbandry and commercial activities [4]. Abuse of antibiotics use in animal husbandry has been highlighted in the country with prevalences of antimicrobial residues of 31% and 51.72% in meat and raw milk, respectively [27, 28]. Although antimicrobial use for animals is under veterinary prescription control in Burkina Faso, farmers still use unprescribed antimicrobials as growth promoters or treatment for cattle, poultry and swine [29, 30]. However, there are similarities between the antibiotics employed in agriculture and veterinary and those prescribed for humans in terms of types and mode of actions [31]. Therefore, consumption of contaminated animal-derived food products by residual antibiotics may pose serious public health concerns. In rural settings of Burkina Faso and in many parts of Africa where there is no enough healthcare facilities [32, 33], a high rate of ESBL producing *Enterobacteriaceae* contaminations is expected among children which might lead to high infantine mortality rates.

## Methods

### Bacterial isolates

**Fig. 1:**
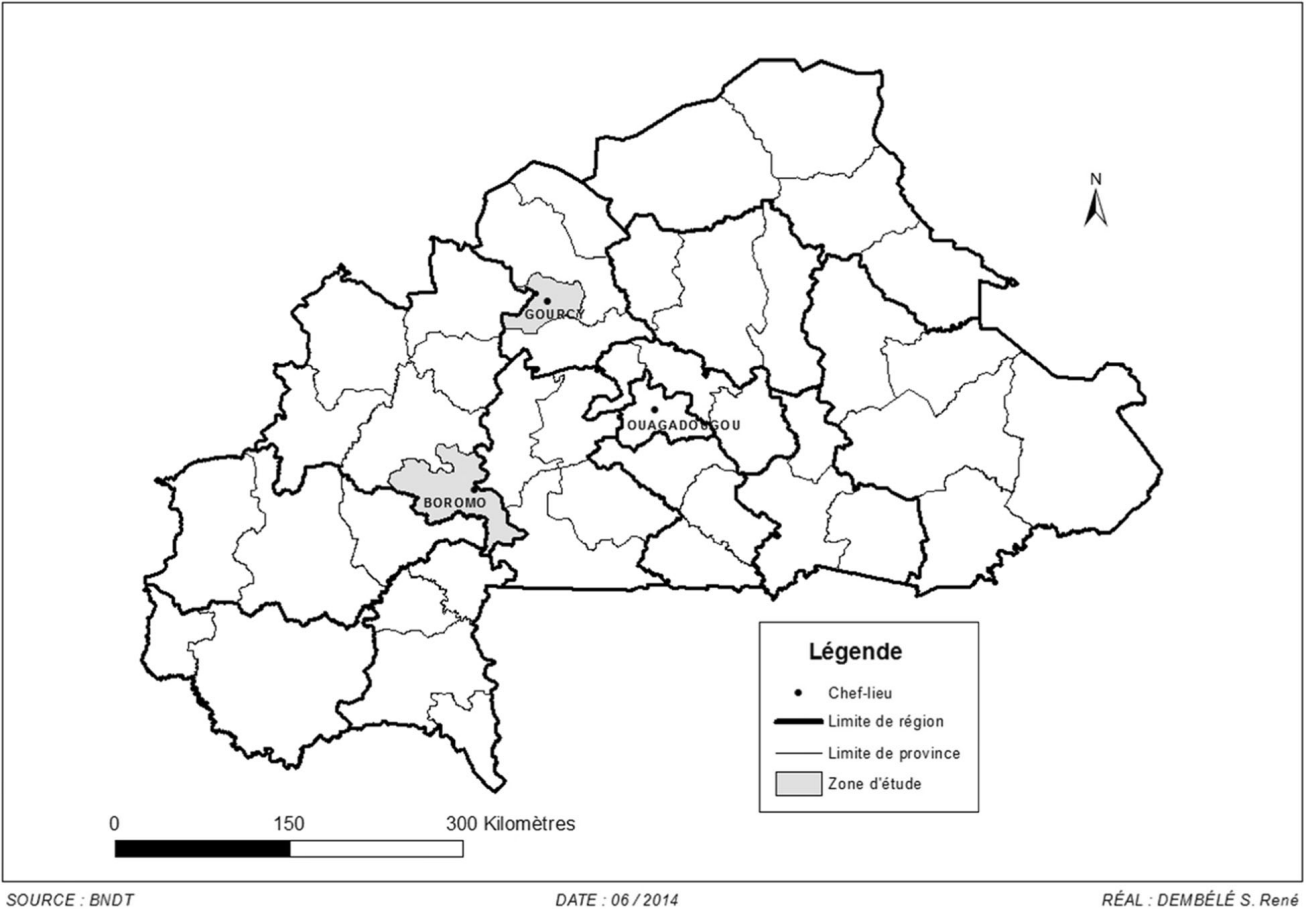
Map of Burkina Faso. In dark = Gourcy and Boromo where the study was conducted. Legend: Software: Quantum GIS (QGIS 2.2), Valmiera, https://qgis.org/downloads/

Strains were obtained from our previous studies [34, 35] conducted in Gourcy and Boromo hospitals’ (Fig. 1). 16-plex PCR was used to detect simultaneously 16 genes from the five main pathogroups of *E. coli* (enterohemoragic *E. coli*: EHEC, enteropathogenic *E. coli*: EPEC, enteroaggregative *E. coli*: EAEC, enteroinvasive *E. coli*: EIEC and enterotoxigenic *E. coli*: ETEC) [36]. Furthermore, all *Salmonella* isolates were serotyped with the somatic O and flagellar H anti-sera according to the Kauffman-White scheme [37].

### Antimicrobial susceptibility test and ESBL production

Antibiotic susceptibility was determined on Mueller-Hinton agar using the standard disc diffusion procedure as described by the European Committee of Antimicrobial Susceptibility Testing (EUCAST) [38]. Nineteen antibiotics belonging to 7 different families were tested as shown in Table 1 (Bio-Rad, France). The diameters of the antibiotic sensitivity halos were recorded according to the EUCAST recommendations. Intermediate (I) susceptibility of pathovars was classified as resistant (R). A double synergy test was used for ESBL-producing strains testing. This consisted of placing discs (2–3 cm diameter) of ceftriaxone and cefotaxime around an amoxicillin-clavulanic acid disc on the bacterial plate.

**Table 1:** Zones of inhibition of the tested antibiotics

**Families**		**Antibiotics**	**[C]**^**a**^ (µg)	**Ǿ** ^**b**^**(mm)**	
				**R (Ǿ˂)**	**S (Ǿ≥)**
β-lactams	Aminopenicillins	Amoxicillin- clavulanic acid (AMC)	30	19	19
		Amoxicillin (AMX)	25	19	19
		Piperacillin (PIP)	75	17	20
		Piperacillin-tazobactam (TZP)	100/10	17	20
	Cephalosporins C3G	Ceftriaxone (CRO)	30	20	23
		Cefixime (CFM)	10	17	17
		Cefotaxime (CTX)	30	17	20
	Cephalosporines C4G	Cefepime (FEP)	30	21	24
	Monobactam	Aztreonam (ATM)	30	21	24
	Carbapenemes	Imipenem (IPM)	10	16	22
Quinolones		Nalidixic acid (NAL)	30	14	19
Fluoroquinolones		Ciprofloxacin (CIP)	5	19	22
Cyclines		Tetracycline (TET)	30	15	18
Phenicols		Chloramphenicol (CHL)	30	17	17
Sulfamides		Trimethoprim-sulfamethoxazole (SXT)	1.25/23.75	13	16
Polymyxines		Colistin sulfate (CST)	50	15	15
Aminoglycosides		Gentamycin (GMI)	15 (10 IU)	14	17
		Netilmicin (NTM)	10	12	15
		Tobramycin (TMN)	10	14	17

^a ^concentration, ^b ^diameter

### Molecular identification of resistance genes

DNA extraction was performed using heating method [39]. A loopful of bacterial growth from Mueller Hinton agar (Liofilchem, Italy) plate was suspended in 1 ml of sterilized water. The mixture was boiled for 10 min at 100 °C and centrifuged for 10 min at 12,000 rpm at + 4 °C. Supernatant was then collected and used in the PCR reactions as DNA matrices. Multiplex PCR assays were performed to assess ESBL-encoding genes, including *bla*_OXA_, *bla*_TEM_, *bla*_CTX−M_, *bla*_SHV_ and the presence of quinolone resistance genes including *qnrA, qnrB, qnrS* from the quinolone-resistant DEC and *Salmonella* strains. Primers (GeneCust, France) used for these amplifications are described in Table 2. The PCR assays were carried out in a 25 ml reaction mixture, which consisted of 2.5 µl of the supernatant added to 22.5 µl reaction mixture. This mixture contained 5U of Taq DNA polymerase (Accu Power, South Korea), deoxyribonucleic triphosphate (10 mM), buffer GC (10X), MgCl_2_ (25 mM) and PCR primers (10 µM). Thermocycling conditions were as follows: 5 min at + 94 °C, followed by 35 amplification cycles at + 94 °C for 30 s, + 59 ± 4 °C for 60 s and + 72 °C for 60 s with a final extension of + 72 °C for 10 min on a thermal cycler (AB Applied Biosystems). Following PCR, the reaction products were separated using electrophoresis in 1.5% agarose gel (weight/volume), stained with Redsaf solution (Prolabo, France) and visualized under UV light (Gel Logic 200) [39].

**Table 2:** Sequences of primers used

**Genetic resistance factors**	**Genes**	**Primers sequence (5’to3’)**	**Weight (bp)**
β-Lactam genes (*bla*)	*bla*_TEM_	F: ATG AGT ATT CAA CAT TTC CG	1080
		R: CCA ATG CTT ATT CAG TGA GG	
	*bla*_SHV_	F : TTA TCT CCC TGT TAG CCA CC	768
		R: GAT TTG CTG ATT TCG CTC GG	
	*bla*_OXA_	F: ATG AAA AAC ACA ATA CAT ATC	813
		R: AAT TTA GTG TGT TTA GAA TGG	
	*bla*_*CTX−M*_	F: -ATG TGC AGY ACC AGT AAR GT	544
		R: -TGG GTR AAR TAR GTS ACC AGA	
Quinolones genes (*Qnr*)	*qnr*A	F: TCA GCA CAA GAG GAT TTC TC	657
		R: GGC AGC ACT ATT ACT CCC A	
	*qnr*B	F: GAT CGT GAA AGC CAG AAA GG	469
		R: ACG ATG CCT GGT AGT TGT CC	
	*qnr*S	F: ACG ACA TTC GTC AAC TGC AA	417
		R: TAA ATT GGC ACC CTG TAG GC	

## Results

### Antimicrobial resistance

**Fig. 2:**
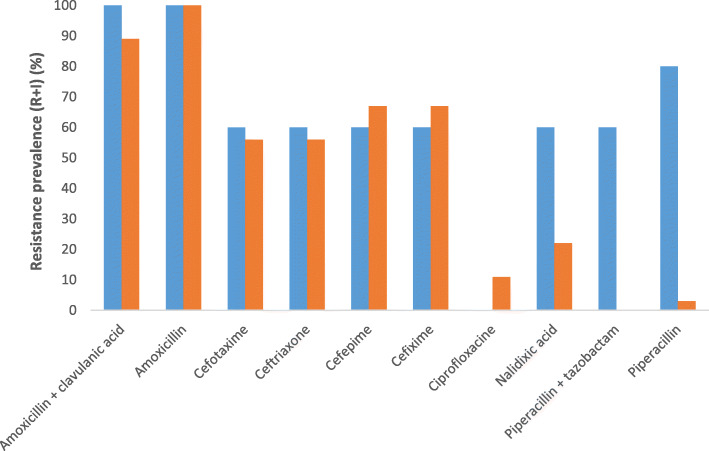
ESBL strains resistance to beta-lactams and quinolones antibiotics. Legend:  = *Salmonella*.  = *Escherichia coli*

At least, 5 diarrheagenic *E. coli* and 9 *Salmonella* strains were identified from our previous studies in 2019 and 2018 respectively. The strains of *E*. *coli* identified exhibited a strong resistance to beta-lactams with 100% resistant to amoxicillin-clavulanic acid and amoxicillin, 80% resistant to piperacillin, 60% resistant to cefotaxime, ceftriaxone, aztreonam, cefixime, cefepime and piperacillin-tazobactam. These strains were less resistant to quinolones, 60% resistant to nalidixic acid and no resistant to ciprofloxacin (Fig. 2). By contrast, the *Salmonella* strains exhibited 100 and 89% resistance to amoxicillin and amoxicillin-clavulanic acid, respectively. Likewise, the resistance of *Salmonella* to cefixime and cefepime, ceftriaxone and cefotaxime were 67 and 56%, respectively. *Salmonella* isolates harboured low resistance to quinolones (22% to nalidixic acid and 11% to ciprofloxacin) (Fig. 2).

### Associated resistance phenotypes

The distribution of the associated resistance phenotypes is shown in Table 3. Extended-Spectrum Beta-Lactamases (ESBL) resistance phenotype was reported in all *E*. *coli* isolates (5/5). Cross-resistance phenotype to quinolones (CRQ) was shown by one *Salmonella* strain (1/9) and three *E*. *coli* (3/5). The Cross-resistance phenotypes to fluoroquinolones (CRFQ) were harboured by one *Salmonella* (1/9) and carbapenemase phenotypes were detected in two *E*. *coli* strains.

**Table 3:** Distribution of *E*. *coli* and *Salmonella* resistance phenotypes and genes

**Isolates**	**Resistance phenotypes**	**Genetic resistance genes**						
		β-Lactam genes				Quinolones genes		
		*bla*_OXA_	*bla*_CTX−M_	*bla*_SHV_	*bla*_TEM_	*qnr*A	*qnr*B	*qnr*S
066B (*S.* Typhimurium)	CRFQ	-	**-**	**-**	**-**	**-**	**-**	**-**
112G1 (*S*. Virchow)	CRQ	-	-	**-**	**-**	**-**	**-**	**-**
084B (*S*. Duisburg)	**-**	+	-	**-**	**-**	**-**	**-**	**-**
057B (*S*. Poona)	**-**		-	**-**	**-**	**-**	**-**	**-**
068B (*S*. Typhimurium)	**-**	+	-	**-**	**-**	**-**	**-**	**-**
078B (*S*. Ouakam)	**-**	+	-	**-**	**-**	**-**	**-**	**-**
063G (*S*. Hvittingfoss)	**-**	-	-	**-**	**-**	**-**	**-**	**-**
087G (*S*. Poona)	**-**	-	-	**-**	**-**	**-**	**-**	**-**
112 G2 (*S*. Virchow)	**-**	-	-	**-**	**-**	**-**	**-**	**-**
025B (EAEC)	ESBL + CRQ	+	-	**-**	**-**	**-**	**-**	**-**
039B (EAEC)	ESBL	+	-	**-**	**-**	**-**	**-**	**-**
043B (aEPEC)	ESBL + Carbapenemase	+	-	**-**	**-**	**-**	**+**	**-**
044B (EAEC)	ESBL + Carbapenemase + CRQ	+	+	**-**	**-**	**-**	**-**	**-**
046B (aEPEC)	ESBL + CRQ	+	-	**-**	**-**	**-**	**-**	**-**

- = absence; + = presence

*SSalmonella*, *EAEC *Enteroagregative *Escherichia coli*, *aEPEC* Atypical Enteropathogenic *E. coli*, *CRQ* Cross-Resistance phenotype to Quinolones, *CRFQ* Cross-Resistance phenotype to Fluoroquinolones, *ESBL* Extended-Spectrum Beta-Lactamases

### Characterization of β-lactamase and quinolones genes

**Fig. 3:**
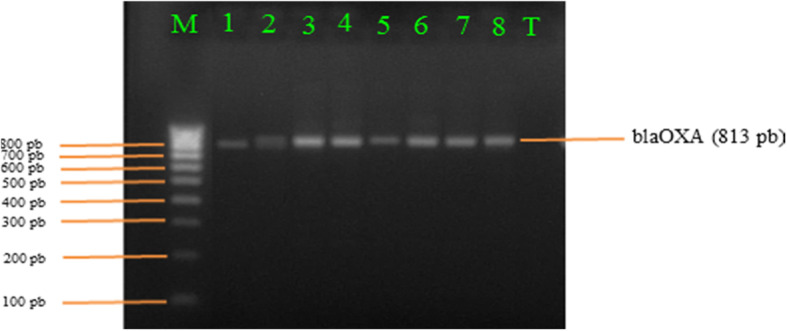
*bla-*_OXA_ gene detected in *E*. *coli*. Legend: Lane M: hyperlader VI (100 bp), Lane 1: *bla*_OXA1_ positive control (813 pb), Lane 2-8: positive samples for *bla*_OXA1_ gene, Lane T: negative sample

Molecular characterization of *E*. *coli* and *Salmonella* isolates revealed that they harboured several ß-lactamase-encoding genes (*bla*_OXA_ and *bla*_CTX−M_). The *bla*_OXA_ genes were detected in 100% (5/5) of *E*. *coli* isolates and in 33.33% (3/9) *Salmonella* isolates (Fig. 3). The *bla*_CTX−M_ gene was detected in one strain of *E*. *coli* and this strain also harboured the *qnr*B gene. The *qnr*A and *qnr*S genes were not detected in any of *E*. *coli* and *Salmonella* strains. The distribution of the different genes encoded is shown in Table 2. The genes *bla*_TEM_, *bla*_SHV_, *qnr*A and *qnr*S were not found in this study.

## Discussion

The current study was undertaken to screen the ESBL and fluoroquinolone resistance genes among *E*. *coli* and *Salmonella* isolated in children with diarrhea in two rural communities of Burkina Faso. Consistent with global reports, an alarming increase in resistance to beta-lactam antibiotics (even to the extended-spectrum subclass) among clinical *E. coli* isolates is highlighted by the results of this study. Indeed, the proportion of resistant strains was 60% for *E*. *coli* and 50% for *Salmonella*. These findings were consistent with previous studies in developing countries which showed a resistant rate greater than 50% [40]. This high resistance is likely due to the extensive and excessive clinical use of antibiotics.

Our study showed that all the *E*. *coli* isolates (5/5) were ESBL producers in agreement with 95.60% reported in Togo [41]. The presence of ESBL-producing bacteria in hospitals is a major challenge that affects both developed and developing countries [26]. It is known that β-Lactams (mainly extended- past spectrum cephalosporins and carbapenems) and fluoroquinolones constitute the main therapeutic choices to treat infections caused by *Enterobacteriaceae*. Although carbapenems are the most effective against Gram-positive and Gram-negative bacteria presenting a broad spectrum of antibacterial activity [42], our findings revealed a strong resistance to beta-lactams and moderate rates of resistance to quinolones in *E*. *coli* and *Salmonella* isolated. Indeed, Cross-resistance phenotypes to quinolones (CRQ), Cross-resistance phenotypes to fluoroquinolones (CRFQ) and carbapenemase phenotypes were associated with different rates to our *Salmonella* and *E*. *coli* strains. In agreement with our results, resistance to these compounds has been reported increasingly in several countries [11, 43, 44], limiting dramatically treatment options. Therefore, older agents, such aspolymyxins and fosfomycin, which were rarely implemented in the past because of efficacy and/or toxicity concerns, together with the newer tige-cycline, have become last-resort choices [42].

According to the existing data, this study is the first of kind on rural samples of Burkina Faso. However, it has been shown that fecal carriage of ESBL-PE isolates is one of the main drivers for their dissemination in hospital and community settings worldwide [45]. Because of this mode of diffusion, the ESBLs constitute a significant threat for the countries of West Africa where `the weak socio-economic conditions result in poor hygienic conditions, promoting the spread of resistance. Indeed, most of the farms in Burkina Faso are mainly traditional with unhygienic practices [27]. Furtheremore, our previous study reported that the people in households mainly use well water as a source of drinking [34].

The present study showed that the *bla*_OXA_ genes were the most common ß-lactamase-producing genes (57.14%), followed by *bla*_CTX−M_ (7.14%). These findings contrast with those previously reported in Burkina Faso [8, 26]. Otherwise, a spread of *bla*_CTX−M_, particularly CTX-M-15 in community and hospital settings has been reported [32, 45, 46]. This difference could be explained by the weakness of the number of multiresistant strains of enterobacteria tested in our study. On the other hand, we noted the simultaneous presence of the *bla*_CTX−M_ and *bla*_OXA_ genes in the same strain of *E*. *coli*. Our finding confirms the frequent association between *bla*_CTX−M−15_ and *bla*_OXA−1_ genes in ESBL-PE isolates which has been reported [45, 47–49]. This coexistence could reduce the therapeutic options for treatment with β-lactam antibiotics. Thus, combined production of CTX-M and OXA enzymes by *E*. *coli* and *K*. *pneumoniae* improved resistance to b-lactamase inhibitors, presumably explaining their non-susceptibility to amoxicillin/clavulanate [45, 49, 50]. The genes *bla*_TEM_ and *bla*_SHV_ were not identified in the present study. In contrast, these genes have been previously reported in three major hospitals of Ouagadougou [26]. A future study based on more multiresistant strains producing ESBL would shed more light on the existence and prevalence of these genes among rural dwellings.

We also reported the prevalence of plasmid-mediated quinolone resistance in *Salmonella* and *E*. *coli*. Only a single isolate of *E*. *coli* (20%) was positive for the *qnr*B gene which is lower than 67.21% reported in Togo [41] and higher than 3.17% reported in Niger [51]. No *Salmonella* strain was positive for the *qnr* genes in the present study. In France, a study revealed 0.2% of *qnr*A in single isolate of *Salmonella* [52]. These results may indicate a low dissemination rate of *qnr* genes among human *Salmonella* and *E*. *coli* isolates. Morever, the *E*. *coli* strain that harbored *qnr*B gene was also positive to ESBL and carbapenemase phenotypes. Indeed, *qnr* are genes that confer resistance to nalidixic acid and reduced susceptibility to fluoroquinolones [53] and there is frequent association of genes coding for expanded-spectrum b-lactamases (ESBLs) and these genes [52].

The main limitation of the present study consists of the low number of isolates which makes generalizability difficult. Further studies consisting of larger sample size than the number of multidrug-resistant isolates considered in the present study would be necessary. Despite this, the results of this study alert us to (i) the emergence and spread of antibiotic resistance in young children, (ii) the existence of *bla* and *qnr* genes in rural areas of Burkina Faso. In addition, the absence of these genes in certain investigated strains maybe due to other mechanisms of resistance to beta-lactams and quinolones.

## Conclusions

This study characterized some *bla* and *qnr* genes circulating in rural settings that are characterized by their easy transfer between bacteria. The results should contribute to the establishment of a surveillance system for antibiotic resistance in Burkina Faso. Indeed, the data gathered is of paramount importance since it may contribute to design strategies to curtail the emergence and spread of ESBL-producing *Enterobacteriaceae* among children in rural Burkina Faso and devise innovative therapeutic approaches against multidrug-resistant strains. The intestinal carriage of ESBL-PE is a significant challenge for public health, and highlights the urgent necessity to improve sanitation and implement antibiotic stewardship in developing countries.

## Data Availability

All data obtained are available within the article.

## Abbreviations

ESBL: Extended-Spectrum Beta-Lactamases
CRQ: Cross-resistance phenotype to quinolones
CRFQ: Cross-resistance phenotypes to fluoroquinolones
ESBL-PE: Extended-Spectrum Beta-Lactamases producing *Enterobacteriaceae*
EUCAST: European Committee of Antimicrobial Susceptibility Testing
